# The Impact of Emotions and Empathy-Related Traits on Punishment Behavior: Introduction and Validation of the Inequality Game

**DOI:** 10.1371/journal.pone.0151028

**Published:** 2016-03-15

**Authors:** Olga M. Klimecki, Patrik Vuilleumier, David Sander

**Affiliations:** 1 Swiss Center for Affective Sciences, University of Geneva, Geneva, Switzerland; 2 Laboratory for the Study of Emotion Elicitation and Expression, Department of Psychology, University of Geneva, Geneva, Switzerland; 3 Laboratory for Behavioral Neurology and Imaging of Cognition, Department of Neuroscience, Medical School, University of Geneva, Geneva, Switzerland; University of Pisa, ITALY

## Abstract

In the prevention and resolution of conflicts in social contexts, an important step is to understand how different emotions and empathic traits are linked to punishment behaviors. Unfortunately, few paradigms exist to study these phenomena. Here, we developed the Inequality Game (IG) as an economic and verbal interaction paradigm in which participants are faced with an “unfair other” as opposed to a “fair other” and subsequently have the opportunity to engage in a range of social behaviors. These social behaviors include cooperative or competitive economic choices and nice or derogatory verbal behavior toward the unfair and fair other. Participants could thus engage in punishment or forgiveness behavior toward the unfair other as well as in cooperative or aggressive behavior toward the fair other. We validated the IG through multimodal measures comprising the assessment of personality traits, emotions (by means of facial expressions and self-reports), arousal (by means of skin conductance responses), physical effort (force exertion), and behavioral reactions. Second, we examined the influence of emotions and empathy-related traits on punishment behavior. With regard to emotions, we observed a positive relation between malicious joy and punishment behavior. This result highlights the role of reward-related mechanisms in favoring punishment behavior. In addition, different empathic traits had opposing effects on antisocial behavior. Whereas personal distress predicted aggressive verbal behavior, perspective taking and empathic concern predicted a reduction in punishment behavior. Empathic traits also modulated emotional experience and person evaluations, such that perspective taking was related to more positive affect (less frowning and more smiling) and a more favorable evaluation of the unfair other. The current data validate the IG, reveal that malicious joy is positively related to punishment behavior, and show that different types of empathic traits can have opposing effects on antisocial behavior as well as on related emotions and person evaluations.

## Introduction

Although the role of emotions and empathic processes in social interactions is increasingly being studied (e.g., [1–4]), many questions remain open regarding their relation to antisocial behavior. This might partly be because extant paradigms for the study of emotional and behavioral responses to aggression (e.g., [5, 6]) do not allow for a clear delineation of reactive as opposed to instrumental (or proactive) aggression [7]. To address these issues, we developed and validated a novel task, the Inequality Game (IG). This task was used to investigate emotional and behavioral reactions to anger-provoking situations and to examine how emotional experiences and empathic personality traits influence these behaviors.

A few experimental paradigms allow for the assessment of behavioral reactions to anger elicitation [5, 6, 8, 9], but they have a number of shortcomings. Anger and aggression can be elicited in the competitive reaction time task [5] and the point subtraction task [6], both of which rely on a temporal structure in which a provocation and the possibility to retaliate are interspersed. In such paradigms, punishment behavior might be driven by reactive aggression, which denotes an affect-driven type of aggression [7], or by instrumental aggression [7], which is less emotional but more oriented towards a goal, such as to change the other’s behavior. Other paradigms relying on humiliation, unjustified reprimands, and insults (e.g., [9]) constitute singular events that are not suitable for concurrently assessing physiological reactions, since the latter necessitates recording data over several trials (such as skin conductance responses [SCRs] or functional magnetic resonance imaging). Still other paradigms from behavioral economics, such as mixed-motive games [8], rely on interdependent payoff matrices in which the choice of one player affects his own and another person's outcome. This structure, however, confounds other-regarding behavior with self-interests. There is thus a shortage of paradigms that at the same time allow for i) the separate assessment of emotional and behavioral reactions to anger elicitation, ii) the concurrent recording of physiological data across repeated trials, and iii) a distinction between self- and other-regarding preferences.

Emotions play a crucial role in guiding judgments and behavior (e.g., [10, 11]). Positive and negative emotions may influence interpersonal exchanges in opposing ways (for review, see [12, 13]). Although different emotions of the same valence can also have opposing effects on decision making (e.g., [14]), positive emotions in general tend to be associated with more cooperation [1], while negative emotions in general and anger in particular have been implicated in intensifying conflict and aggression (e.g., [2, 15]). Although some research has shown that induced positive emotions can promote cooperative behavior (e.g., [1]), there is a shortage of studies investigating the link between emotional experiences, such as anger, with social behavior, such as aggression. Anger and aggression are conceptually linked, although anger does not always lead to aggression [15]. Another emotion that might be of interest here is malicious joy, as it has been linked to punishment [16,17]. Observing an unfair person being punished was thus associated with malicious joy [16] and punishing an unfair person was accompanied by neural activations in reward-related areas [17]. Despite these findings, the relation between emotional experiences, such as malicious joy, and active punishment behavior remains largely unresolved.

Smooth social interactions are greatly facilitated by perspective taking and empathy. Perspective taking is defined as the ability to infer the thoughts and intentions of others through cognitive processes that allow one to simulate the perceptions and beliefs of others [18]. Empathy is commonly defined as the affective sharing and understanding of both pleasant and unpleasant emotions [19]. When it comes to sharing of unpleasant emotions, two components of empathy can be differentiated–empathic distress (also called personal distress, e.g., in [20]) and empathic concern (also called compassion, e.g., in [21] or sympathy, e.g., in [22]). Whereas empathic distress denotes the sharing of others’ suffering to a strong degree, leading to a contagion with negative affect, empathic concern occurs when the suffering of others is met with a more positive feeling of caring and the motivation to help (for details, see [23]). A meta-analysis revealed that in situations that involve no conflict, the capacity for perspective taking was related to prosocial behavior [24]. However, it is unknown whether a similar relation also holds for conditions eliciting anger and bearing the possibility for punishment and aggression. Another meta-analysis revealed that empathy in general was associated with reduced aggression [25]. In situations involving needy others, however, empathic skills may have opposing effects on helping behavior [3]. More specifically, other-oriented empathic feelings, called empathic concern or compassion, are associated with prosocial behavior and positive affect [3, 26], whereas feelings of empathic distress lead to reduced helping behavior and increased negative feelings [3]. In light of these opposing effects of different empathy-related components, an open question is whether empathic distress and empathic concern may also have an opposing impact on emotional and behavioral reactions to anger provocation.

What are potential factors reducing punishment behavior and aggressive response following anger elicitation? Previous research has shown that the experience of positive affect is usually associated with more cooperation [1, 12, 13]. However, it remains an open question as to whether strengthening other-related positive affect can also reduce punishment behavior. Other results suggest that the opportunity to “settle the score,” i.e., to engage in revenge behavior, can reduce subsequent aggression [27]. This contrasts with the finding that engaging in aggressive actions can prompt more aggression (e.g., [28]). In light of these opposing findings, more research on the effects of "settling the score" is needed.

The present study addressed these issues by designing and validating the Inequality Game (IG) as a novel paradigm with several complementary measures. Our first aim was to develop a task that can dissociate emotional and behavioral responses to anger elicitation, and allows for the concurrent recording of physiological data. To validate this task, we expected to find evidence for a reliable engagement of participants during the game, as reflected not only by self-reports and behavioral ratings (prediction 1a), but also by physiological measures related to arousal (quantified by SCRs [29, 30]) and effort (quantified by handgrip force [31, 32]) (prediction 1b). We also expected that the IG would induce anger (correlating with individual trait anger), punishment behavior in economic exchanges, and unfavorable person evaluation for the unfair other (prediction 1c). Finally, we expected that the IG would allow for the classification of participants in different types (prosocial, sanctioning, or competitive) based on their behavior (prediction 1d).

A second aim of our study was to more specifically examine the relationship between emotions and punishment behavior. We tested whether emotional expressions (e.g., facial expressions or self-reports) could predict subsequent behavioral reactions towards fair and unfair others (prediction 2a). Spontaneous facial expressions were recorded to probe for displays of happiness (as indexed by smiles) and negative affect (as indexed by frowns). Previous work with the Facial Action Coding System [33] established that the activity of the zygomaticus muscle (smile) is indicative of happiness, while the activity of the corrugator muscle (frown) is associated with unpleasant emotions such as anger, sadness, or fear [34]. In addition, we explored how more specific emotional experiences, such as anger, malicious joy, and regret, were linked to punishment behavior. These indices allowed us to test whether aggression is primarily related to negative emotions, such as anger [2], and to explore whether reward-related emotions, such as malicious joy, may also play a role in driving punishment behavior (question 2b).

The third aim of our study was to examine whether–in analogy to findings from the domain of helping behavior [3, 24]–different empathy-related skills can impact punishment behavior in opposing ways. More specifically, we predicted that empathic concern and perspective taking would be related to a reduction in punishment behavior, while the tendency to experience personal distress would be related to more frequent punishment behavior (prediction 3a). In addition, we explored how emotions, empathy-related traits, person evaluation, and social behavior are linked to each other in the context of anger provocation (question 3b).

A final aim was to investigate factors that may promote a reduction in punishment behavior. To this end, we examined two possibilities. First, we hypothesized that strengthening positive other-related emotions prior to playing the IG would reduce subsequent punishment behavior (prediction 4a). Second, we wanted to test two competing hypotheses: would "settling the score" in one situation (the IG) indeed reduce subsequent punishment behavior in another task, or would there still be significant carry-over effects to a different situation (question 4b)? To this end, we included a seemingly unrelated task subsequent to the IG in which participants could assign desirable and undesirable food items to their former interaction partners.

In summary, the current work allowed us to establish the validity of the IG and its usefulness for obtaining a range of different measures (including self-reports, physiological data, and behavioral data) in relation to anger and social conflict. In addition, our results shed new light on the relation between emotions, empathy-related traits, and punishment behavior.

## Materials and Methods

### Participants

The final sample consisted of 40 men who were mainly students (age: *M* = 24.78 years; *SD* = 5.34; range: 18–37). Six of the initial 46 participants were excluded, as they saw through the nature of the manipulation. We restricted our sample to male participants because of previously reported sex differences in aggression [35, 36] and emotional responses to unfairness [16], with the aim to ensure that we observed a range of aggressive responses in this initial validation study. Prior to the experiment, volunteers completed online questionnaires concerning demographic information, alexithymia (Toronto Alexithymia Scale [TAS-20] [37], French version [38]), and depression (Beck’s Depression Inventory [BDI-II] [39]). Volunteers were included if they met the following criteria: male, aged 18–40, TAS score ≤ 60, and BDI score ≤ 28. Participants provided informed written consent, received 25 CHF for their participation, and were fully debriefed after the experiment. The study was approved by the Research Ethics Committee of the Faculty of Psychology and Educational Sciences at the University of Geneva.

### Measures

#### Inequality Game

The IG was developed to separately assess emotional and behavioral reactions to anger provocation in a socio-economic interaction. It was programmed by using Cogent 2000 (developed by the Cogent 2000 team at the FIL and the ICN and Cogent Graphics developed by John Romaya at the LON at the Wellcome Department of Imaging Neuroscience, London, UK). Participants were told that they played the game with two other participants. Unbeknownst to them, the behavior of the two other players was preprogrammed to be fair or unfair. To increase credibility, participants could freely interact with these two ostensible other participants prior to the testing. At this point, the two confederates did not know whether they would be assigned the fair or unfair role during the IG.

The paradigm had a 2 x 2 x 2 within-subject design with the following three factors: the power of the participant (low power in the first phase of the game, followed by high power in the second phase of the game), the behavior of the other (fair or unfair), and the event type during the interaction (economic interaction or verbal feedback).

**Fig 1:**
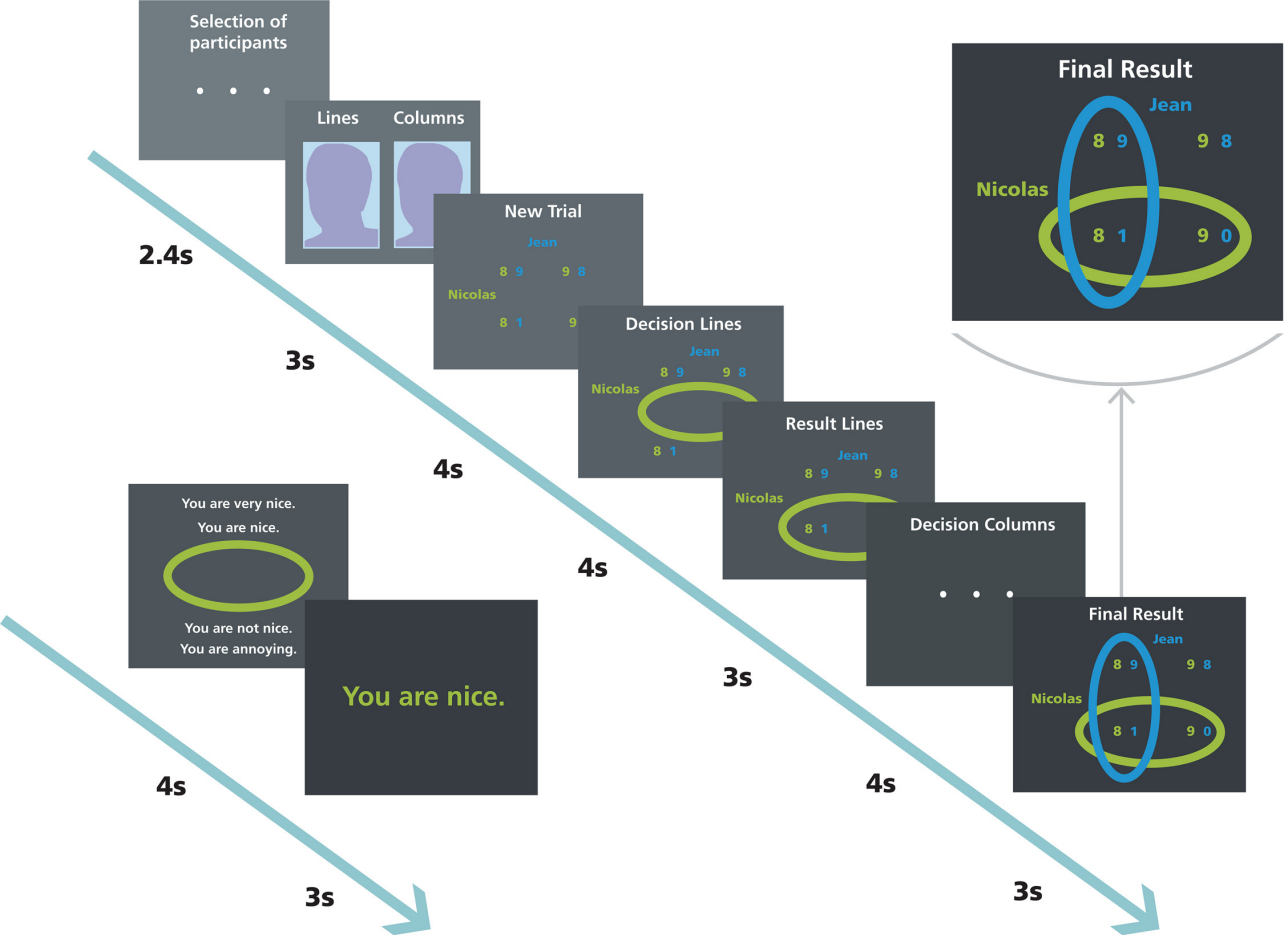
Trial timeline of the Inequality Game, depicted for the high power phase. After player selection, the photographs of both paired players were presented, along with their assignment to control the lines (high power) or the columns (low power). The payoff structure of a new trial was shown for 4 s. The participant (here Nicolas, in green) then chose one of the two lines. After displaying the participant’s choice, the ostensible other (here Jean, in blue) made a decision. The common choice (Final Result) determined the payoff (here: 8 CHF for Nicolas and 1 CHF for Jean). Half of the economic interactions were followed by feedback trials. Here, the player with high power (green) had the opportunity to select between one of four feedback choices for the other, which were subsequently displayed. In the low power phase, the participant was in control of the columns (blue).

The first phase of the game served to assess the participant’s emotional reactions to anger elicitation. Therefore, the participant was in low power and the two others (the fair and unfair other) were in high power. In each trial of the game (Fig 1), the participant was paired with either the fair or with the unfair other. During economic events, the participant interacted with one of the two others to make a common decision on a 2 x 2 payoff matrix that determined the payoff of both players (the participant and the other). The player in high power controlled the lines and chose first. The player in the low power condition controlled the columns and chose second. The player in high power affected up to 90% of the other’s gain in a given trial. He could, for instance, choose between a cooperative distribution (e.g., both interaction partners receive 9 or 10 CHF) and a competitive distribution (e.g., 9 or 10 CHF for oneself and 1 or 0 CHF for the other). Conversely, the player in the low power condition could affect only up to 10% of the gain difference in a given trial (corresponding to differences of 1 CHF). The game was designed in such a way that the fair other selected cooperative outcomes, thereby maximizing both his own and the participant’s benefits, whereas the unfair other chose competitive outcomes that maximized his own benefits while minimizing the participant’s benefits.

We implemented another more social interaction by ensuring that half of the economic choices were followed by a trial in which the player in high power could select one of four feedback messages for the other. In the low power phase, the fair other always sent nice feedback messages to the participant (“You are very nice” three times and “You are nice” three times). Conversely, the unfair other always sent derogatory feedback messages to the participant (“You are not nice” three times and “You are annoying” three times).

In the subsequent phase of the IG, the participant was in turn given high power, allowing us to probe for the participants’ behavioral reactions to anger provocation. In this phase, participants controlled the economic distributions for the fair and unfair other and selected one of the four feedback messages for the fair and unfair other. Participants could thus choose between cooperative or competitive economic distributions for the others and between nice or derogatory feedback sentences for the others.

In total, each phase (low and high power) consisted of 24 economic choices and 12 feedback choices, which were equally split into interactions with the fair and unfair other. Twelve of the 24 payoff matrices reflected situations in which the high power other chose between a cooperative and a competitive allocation of money for the other (e.g., 8/9, 9/8 or 8/1, 9/0 CHF). In order to control for the monetary reward, we ensured that the remaining 12 trials had essentially predetermined economic outcomes (win or no win). These control conditions consisted of six win trials (e.g., 10/9, 10/10, 9/10, 9/10 CHF) and six no win trials (e.g., 0/1, 1/1, 0/0, 1/1 CHF). When the fair and unfair other were in low power or during the control conditions (win, no win for both players), their choices always served to maximize their own gain.

In contrast to mixed-motive games [8], the high power player’s payoff in the IG was largely independent of the low power player’s payoff and thus reflects other-regarding choices. Following the classification of persons as prosocial when they maximize joint outcomes and competitive when they maximize their relative advantage over other’s outcomes [40, 41], the IG allows for the classification of participants as prosocial, sanctioning, and competitive. Participants were identified as prosocial if they chose more cooperative than competitive economic outcomes for both the fair and unfair other. They were classified as sanctioning if they reciprocated by choosing more cooperative than competitive economic outcomes for the fair other and more competitive than cooperative outcomes for the unfair other. Finally, they were classified as competitive if they chose competitive outcomes more often than cooperative outcomes for both the fair and unfair other, thereby maximizing the relative difference between their own and the other’s economic gain.

Participants’ economic and feedback behavior in the high power phase of the IG was operationalized as follows. Economic behavior was quantified by subtracting the number of competitive economic choices from the number of cooperative economic choices, yielding an indicator of cooperation (positive values) as opposed to competition (negative values). In order to test the effect of participants’ choices on economic outcomes, the sum of hypothetical gains from the high power phase was computed for all three players. Feedback behavior was quantified by computing the sum of weighted frequencies with which participants chose each of the four feedback sentences (“You are nice” was assigned a weight of +2, “You are nice” +1, “You are not nice” -1, and “You are annoying” -2). The feedback index, which was separately computed for the fair and unfair other, thus reflects the degree to which participants chose nice (positive value) as opposed to derogatory messages (negative value). Overall, participants’ behavior in the high power phase towards the *unfair* other can be interpreted as punishment behavior (competitive economic choices and derogatory behavior) or forgiveness behavior (cooperative economic choices and nice feedback), whereas social behavior towards the *fair* other is indicative of aggressive behavior (competitive economic choices and derogatory feedback) or prosocial behavior (cooperative economic choices and nice feedback).

#### Skin Conductance Responses (SCRs)

To obtain an objective measure of emotional arousal, skin conductance responses (SCRs) were continuously recorded during the IG by means of two electrodes attached to the index and middle finger of participants’ non-dominant hand (the handgrip device was held with their dominant hand). Data were collected with the MP150 Biopac System (Santa Barbara, CA, USA) and analyzed offline with Acqknowledge 4.1 (Goleta, CA, USA). Because of technical problems, we had no SCR data from five participants, resulting in a final sample size of *n* = 35 for SCR data. SCRs were low-pass filtered at 0.25 Hz and high-pass filtered at 0.05 Hz. SCRs were extracted for each frame of the IG (see Fig 1) and each experimental condition. Our analysis focused on SCRs at the time of the high-power player’s economic or feedback choice. Amplitudes of SCRs that began between 1 and 3 s after stimulus onset were taken into account if they exceeded a threshold of 0.01 μS. Amplitudes were log-transformed by using the formula *A*_ln_ = ln(*A* + 1).

#### Effort

To obtain an objective measure of motivational involvement during the IG, participants were required to make their behavioral choices (with their dominant hand) by using an isometric handgrip device (TDS121C), connected to the MP150 Biopac System (Santa Barbara, CA, USA). Prior to the experiment, we calibrated the baseline (participants were merely holding the device) and the maximal force with which participants could press the device. For data analysis, the data points were converted to the percentage of each participant’s maximal grip force (% of maximum).

#### Facial Expressions

To assess spontaneous emotional reactions, participants’ facial expressions were recorded throughout the IG by using a Logitech USB HD Pro web camera C920 (Apples, Switzerland) mounted on the computer screen. Video recordings were synchronized with the physiological recordings through the MP150 Biopac System (Santa Barbara, CA, USA). Because of technical problems, video data from two participants were not properly recorded. This resulted in a sample size of *n* = 38 for facial expression data. The Computer Expression Recognition Toolbox [42] was used to quantify the intensity of smiles and frowns, indexing positive and negative affect, respectively. Based on the Facial Action Coding System [33], this program extracts the intensity of several facial action units (AUs) from video data. Past research [33] suggests that activity of the zygomaticus muscle (smile) is indicative of happiness, while activity of the corrugator muscle (frown) is associated with negative emotions such as anger, sadness, or fear [34]. Therefore, we used data from AU 12 (lip corner puller, zygomaticus major, smile) and AU 4 (brow lowerer, corrugator, depressor glabellae, depressor supercilii, frown) for our analyses. We smoothed and thresholded the data obtained from the Computer Expression Recognition Toolbox with a custom made toolbox from our laboratory.

#### Food Allocation Task

To assess the propensity to punishment or retaliation following anger provocation, subsequent to the IG, participants were invited to take part in another experiment which was presented as unrelated but done with the same volunteers for convenience. This experiment was described as a food evaluation task in which all three participants had to allocate, taste, and evaluate different food items in separate rooms. The food allocation task (for a detailed description, see see Supporting Materials and Methods in Text A in S1 File) was used to examine the extent of prosocial or aggressive behavior (amount of allocated chocolate sauce and wasabi, respectively) in a novel situation. With the addition of prosocial behavior, the present paradigm can thus be seen as an extension of the hot-sauce paradigm [43]. After this task, the aim of the entire experiment was revealed and neither the participant nor the two confederates actually tasted any food items.

#### Questionnaires

To assess the validity of the IG in relation to well-known individual differences, we asked participants to fill in the following online questionnaires prior to the experiment: the State-Trait Anger Expression Inventory (STAXI-II [44], French version [45]), the Aggression Questionnaire (AQ [46], French version [47]), and the Levenson Self-Report Psychopathy Scale [48], French version [49]). As previous studies found that questionnaire measures of physical and verbal aggression were positively related to behavioral activation (behavioral approach) and negatively related to behavioral inhibition (aversive motivation) [50], we also included the Behavioral Inhibition System/Behavioral Activation System (BIS/BAS) scales [51] (French version [52]). To test for dimensions of empathy, we included the Interpersonal Reactivity Index (IRI [20], French version [53]). Questionnaires were collected prior to the experiment by using the online program Questionnaire Machine (developed by Christoph Hofstetter, University of Geneva). Table A in S1 File summarizes the psychometric characteristics of the sample. Information on the subscales of the questionnaires can be found in the Supporting Materials and Methods in Text A in the S1 File.

During the experiment itself, participants also filled in additional questionnaires (all using 11-point response scales ranging from 0, not at all, to 10, extremely). In order to inform participants that the two others disliked spicy food, we administered a food preference questionnaire at the beginning of the experiment. It asked “How much do you like the following flavors?” in relation to four items: sweet, salty, mild, and spicy. When filling in this questionnaire, the two alleged other participants explicitly stated that they dislike spicy food and fear eating it in the food tasting part of the experiment. Emotions in relation to the IG (joy, anger, disappointment, and sadness) were assessed with a self-report questionnaire depicting instances of the fair or unfair others' economic choices and feedback messages. The self-report questionnaire also assessed self-reported emotions (anger, joy, disappointment, regret, culpability, malicious joy, generosity, and sadness) related to the participants’ own choices (cooperative or competitive economic decisions, as well as nice or derogatory feedback messages to the fair and unfair other, respectively). Self-report questionnaires on emotions were administered after the IG because previous studies observed that explicit emotion assessments can reduce subsequent aggressive behavior [54].

Finally, after the food allocation task, participants were given a debriefing questionnaire that probed for their involvement in the economic interaction game, the extent to which they felt the presence of the others during the game, and their impression of the others as fair, agreeable, good-looking, and reliable. This questionnaire also asked participants about their strategy during the economic interaction game, whether they took the food preferences of the others into account when allocating the food items, and what they thought about the experiment’s aim. These data served as additional information on whether participants believed that the interactions were real.

#### Analyses

Statistical analyses were performed by using SPSS Statistics 21 software. We conducted repeated measures multivariate analyses of variance (MANOVAs), as well as analyses of variance (ANOVAs) and *t*-tests. Correlations between normally distributed variables were tested with Pearson correlations (denoted by *r*) and correlations with variables that were not parametrically distributed were tested with Spearman correlations (denoted by *rs*) on rank transformed data.

## Results

### Aim 1: Validation of the IG

#### Validity of the IG

Detailed results related to the validity of the IG can be found in Text A in the S1 File. In line with prediction 1a, the validity and efficacy of our paradigm was supported by participants’ self-reports of high involvement (*M* = 7.45, *SD* = 1.71) and reports of feeling the others’ presence during the game (*M* = 6.23, *SD* = 2.48; both measured on a scale from 0, not at all, to 10, extremely). This was further supported by higher arousal responses (SCRs) and stronger physical effort (grip force) in reaction to others’ deliberate economic choices, in comparison to control conditions lacking social relevance (Fig A, Table B and Text A in S1 File), in line with prediction 1b.

#### Emotions Elicited by the IG

**Fig 2:**
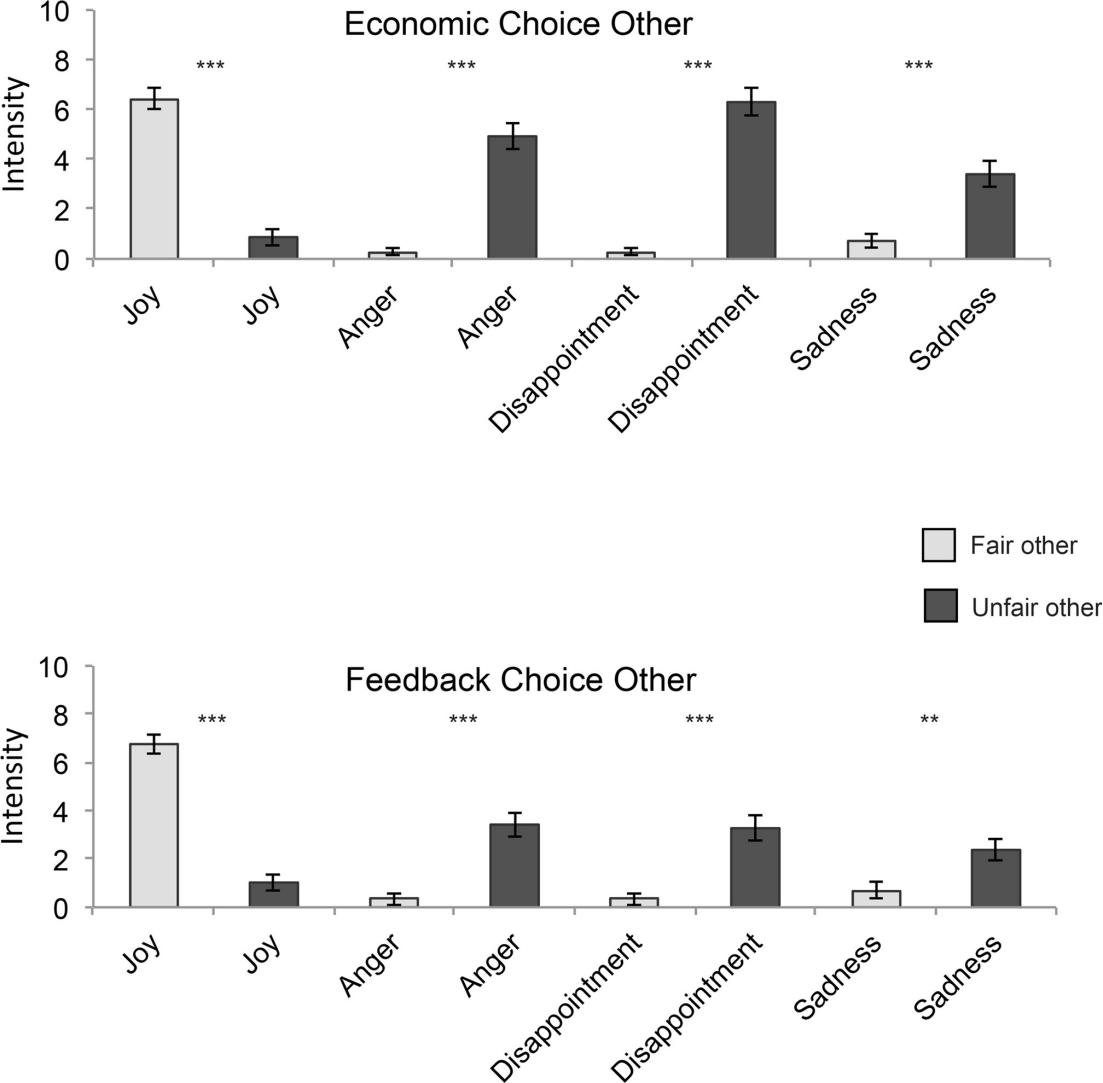
**Self-reported feelings in response to the fair other’s and the unfair other’s economic choices (A) and feedback choices (B) during the low power phase of the Inequality Game.** Charts depict means and 2 standard errors of the mean. ***p < .001; **p < .01.

Similarly, self-reports of emotions also supported our prediction 1c, with higher ratings of anger in response to the unfair other´s behavior in the low-power phase (Fig 2). In addition, the unfair other´s behavior induced stronger feelings of disappointment and sadness, whereas the fair other´s behavioral choice elicited more joy (for details, see Text A in S1 File).

The external validity of the IG as a paradigm to elicit anger and aggression was confirmed by correlations with questionnaire data pertaining to personality traits related to anger and aggression (details in Text A in S1 File). Anger in response to competitive economic choices correlated with trait aggression (AQ; *rs* = .41, *p* < .01) and angry temperament (STAXI-T; *rs* = .33, *p* < .05). Persons who had a tendency to show angry reactions to provocation in their everyday life (STAXI-R) made more competitive economic choices for the fair and unfair other (both *rs* ≥ .33, both *p* < .05) and chose more derogatory feedback for the fair and unfair other (both *r* ≥ .36, both *p* < .05). Details on participants´ emotional experiences related to the high-power phase and the difference of emotions in the high- and low-power phase can be found in Text A and in Tables C-E in S1 File). Finally, in line with prediction 1c, the behavior of the fair and unfair other during the IG led to a more favorable evaluation of the fair other (Text A and Table F in S1 File)–despite counterbalancing confederates to the fair and unfair condition.

#### Reciprocation of Fair and Unfair Behavior

**Fig 3:**
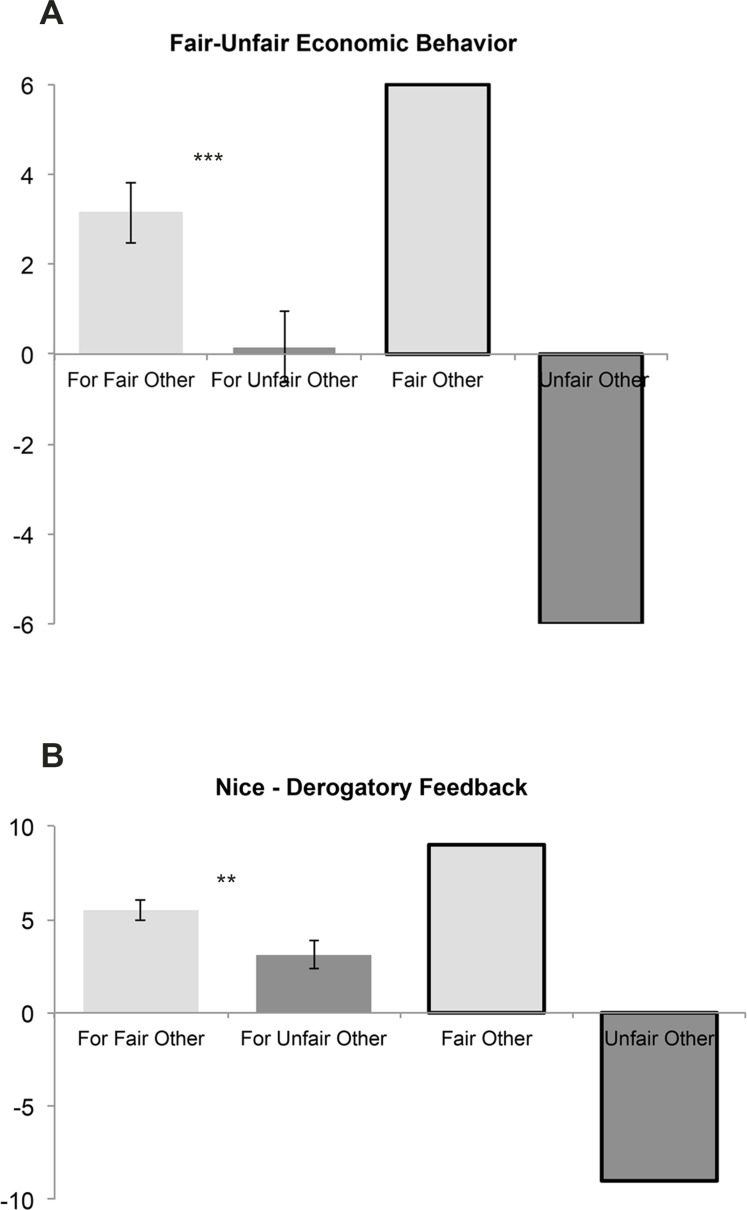
Participants’ behavior in the high power phase of the Inequality Game. Participants’ economic choices (A) and feedback choices (B). For completeness, the corresponding behavior of the fair and unfair other in the low power phase is displayed on the right-hand side of each plot. Charts depict means and 2 standard errors of the mean. ***p < .001; **p < .01.

To test for the behavioral impact of the fairness manipulation in the low-power phase, we assessed whether participants displayed differential behavior towards the fair and unfair other during the subsequent high-power phase. A repeated-measures MANOVA was performed with the within-subject factor other (two levels: fair and unfair), the between-subject factor focus (positive qualities or physical description), and the two dependent variables, economic choice and feedback choice. This revealed a large effect of other (*F(2*,*37)* = 10.98, *p* < .001, *ƞ*^*2*^ = .37), while the effect of focus was not significant (*F(2*,*37)* = 0.16, *p* = .86). Confirming prediction 1c on the behavioral level, univariate ANOVAs showed that both economic choices (*F(1*,*38)* = 15.69, *p* < .001, *ƞ*^*2*^ = .29) and feedback choices (*F(1*,*38)* = 13.17, *p* < .01, *ƞ*^*2*^ = .26) were more favorable for the fair other than for the unfair other (Fig 3).

To further test whether participants’ economic and feedback behavior towards the fair and unfair other differed from the respective behavior of the alleged others, we used one-sample *t*-tests. These analyses revealed that, for both economic choices and feedback messages, the participants’ behavior was less extreme than the behavior of the respective other (i.e., less prosocial then the fair partner and less antisocial than the unfair; all *t*(39) ≥ 4.22; all *p* < .001, Fig 3).

Finally, we found that economic choices and feedback choices were positively correlated. This was the case for behavior towards both the fair and the unfair other (both *rs* ≥ .38, both *p* < .05), indicating that participants’ behavior was consistent across event types.

#### Classification of Participants Reveals Preference for Conciliatory Behavior

**Fig 4:**
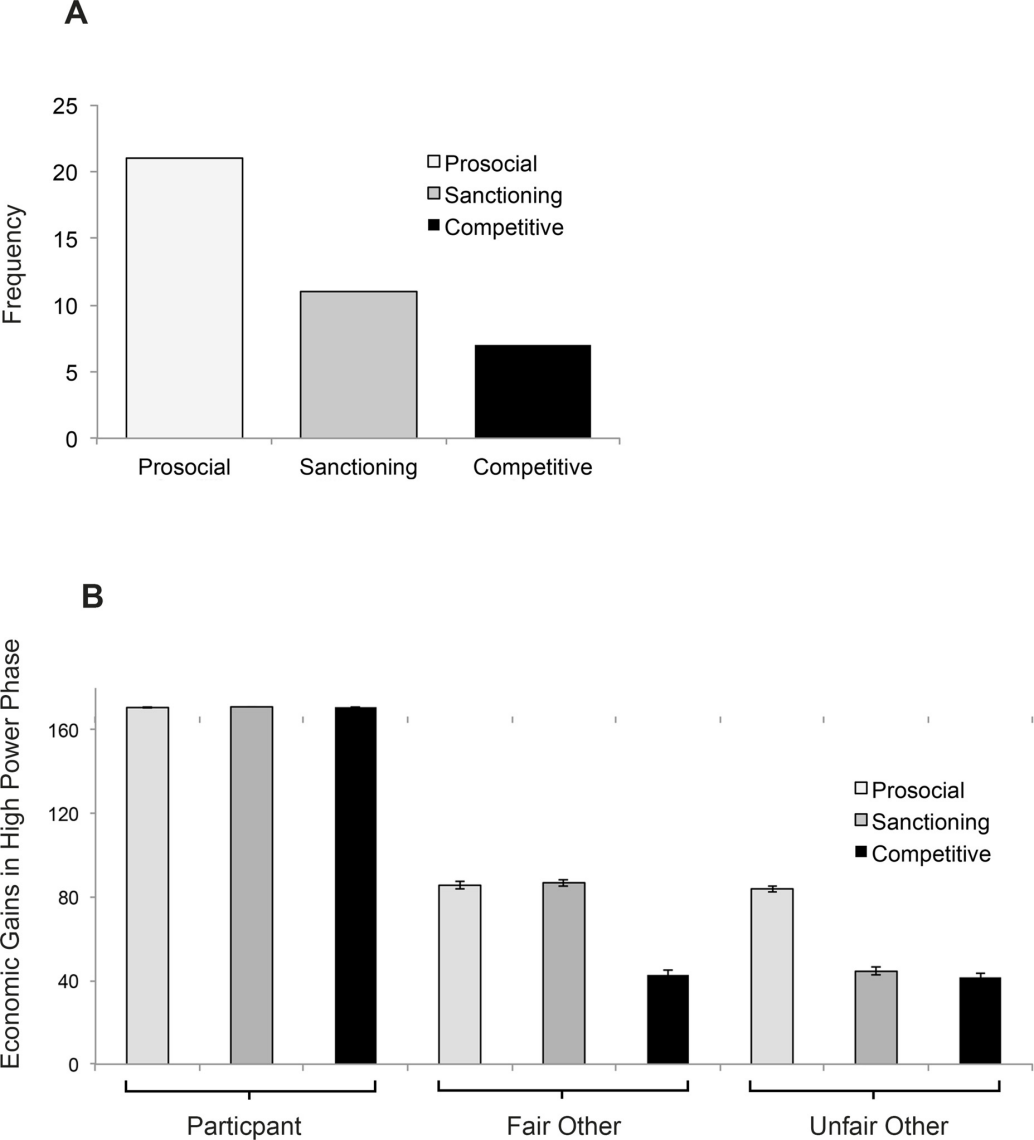
Participants’ behavior in the high power phase of the Inequality Game. (A) Classification of participants based on their economic choices in the high power phase and (B) hypothetical economic gains in the high power phase for each player split by inter-individual classification of participants. Chart A depicts frequencies; Chart B depicts means and 2 standard errors of the mean. ***p < .001; **p < .01.

On the basis of data from the high-power phase, participants could be categorized according to their preferences for cooperative, sanctioning, or competitive economic behavior (Fig 4A; see methods and prediction 1d). In our sample, 21 of 40 participants were prosocial, 11 sanctioning, 7 competitive, and 1 ambiguous (no systematic preference). As detailed in the results section of Text A in the S1 File, the independent payoff structure of the IG was corroborated, as participants’ behavioral preferences for prosocial, sanctioning, or competitive behavior affected only the others’ economic outcomes, while participants' own payoffs remained unaffected by these preferences (Fig 4B). Furthermore, participants with distinct classifications also differed in terms of anger-related personality traits (detailed in Text A in S1 File).

Using binomial tests, we determined whether participants’ actual behavior deviated from behavior predicted by chance distribution. These tests revealed that despite exposure to unfairness and insults in the low power phase, more participants than expected by chance preferred to engage in prosocial behaviors towards both the fair and unfair other in the subsequent high power phase. This was the case in terms of cooperative economic choices (*p* < .01) and nice feedback messages (*p* < .001).

### Aim 2: Relationship between Emotions and Behavior

To examine the relation between emotions and behavioral reactions to anger provocation, we used several measures, including spontaneous displays of positive and negative affect during the provocation phase and self-reports of specific emotional experiences in particular situations of the IG. This allowed us to obtain both implicit and explicit measures, probing general valence and more specific emotions, respectively.

#### Facial Expressions Related to Pleasant and Unpleasant Emotions

To test whether a spontaneous display of happiness (with a smiling facial expression) as opposed to unpleasant emotions (with a frowning facial expression) during the provocation phase may predict subsequent behavioral reactions, we correlated the intensity of participants’ smiles and frowns during the low power phase with their economic and feedback choices in the subsequent high power phase. In line with our hypothesis (prediction 2a) that positive emotions are linked to prosocial behavior, the average smile intensity during the low power phase predicted fair economic choices towards the unfair other in the high power phase (*rs* = .28, *p* < .05, one-tailed). No such effect was found for frowns (*rs* = -.2, *p* = .22). There was no relation between the display of frowns or smiles in the low power phase and feedback behavior in the high power phase (all *r* ≤ .1, all *p* ≥ .56).

#### Relation between Self-Reported Emotions and Behavior

We also assessed whether specific emotional responses during the IG were related to different behaviors (prediction 2b). In the low power phase, self-reported feelings (joy, anger, disappointment, and sadness) evoked by others’ economic and feedback behavior, were not correlated with subsequent economic or feedback behavior in the high power phase (all *rs* ≤ .25, all *p* ≥ .13). In contrast, behavioral choices in the high power phase were correlated with some of the associated self-reported feelings. When participants chose cooperative monetary outcomes for the fair other, stronger feelings of regret were related to less cooperative economic behavior (*rs* = -.34, *p* < .05). Similarly, stronger feelings of disappointment were related to less frequent nice feedback choices for the fair other (*rs* = -.4, *p* < .05).

When participants interacted with the unfair other, self-reports of malicious joy during punishment behavior correlated with the number of competitive economic choices (*rs* = .4, *p* < .05). Similarly, malicious joy experienced during the delivery of derogatory feedback correlated with the degree to which such feedback was given (*rs* = .44, *p* < .05). Self-reports of joy pointed in the same direction (*rs* = .37, *p* < .05), while the experience of regret was negatively related to the amount of derogatory feedback given (*rs* = -.4, *p* < .05). All other correlations were not significant (all *rs* ≤ .29, all *p* ≥ .07), including the relation between state anger and punishment behavior. A formal comparison of correlation coefficients with Williams’ *t*-test revealed that malicious joy was more strongly related to competitive economic behavior (*t(27) =* 2.53, *p* < .05) and derogatory feedback choices (*t(26) =* 2.83, *p* < .01) towards the unfair other than anger was.

### Aim 3: Relationship between Empathy-related Traits and Behavioral Reactions to Anger Provocation

To examine the role of empathy, we tested whether empathic traits measured by the IRI subscales perspective taking, empathic concern, and personal distress were differentially related to participants’ behavior during the high power phase of the IG and during food allocation (prediction 3a). As expected, the capacity for perspective taking (e.g., "When I’m upset at someone, I usually try to ‘put myself in his shoes’ for a while") predicted nice feedback choices for the unfair other (*r* = .39, *p* < .05) and cooperative economic choices for the unfair other (*rs* = .32, *p* = .05). Perspective taking had no relation to behavior towards the fair other. In contrast, personal distress (e.g., "Being in a tense emotional situation scares me") predicted derogatory feedback towards both the fair and unfair other (*r* ≥ .37, *p* < .05). Empathic concern had no relationship with choices in the IG.

With regard to food allocation, empathic concern (e.g., "I often have tender, concerned feelings for people less fortunate than me") predicted how much desirable food (chocolate sauce) participants allocated to the unfair other (*rs* = .46, *p* < .01). Perspective taking and empathic distress showed no significant correlation with behavior in this task.

These findings show that when anger is induced, persons high in perspective taking and empathic concern displayed less punishment behaviors. Conversely, people prone to experience distress displayed more verbal aggression, even towards fair others. The link between the experience of distress and antisocial behavior is further corroborated by the observation that the higher participants scored on behavioral inhibition (BIS; e.g., “Criticism or scolding hurts me quite a bit”), the more likely they were to send derogatory feedback to the fair and unfair other (both *r* ≥ .45, both *p* < .01). With regard to prediction 3b, perspective taking correlated with more positive affect (less frowning, more smiling) and more favorable evaluation of the unfair other (all *p* < .05; see details in the results of Text A in S1 File).

### Aim 4: Factors Reducing Punishment Behavior

**Fig 5:**
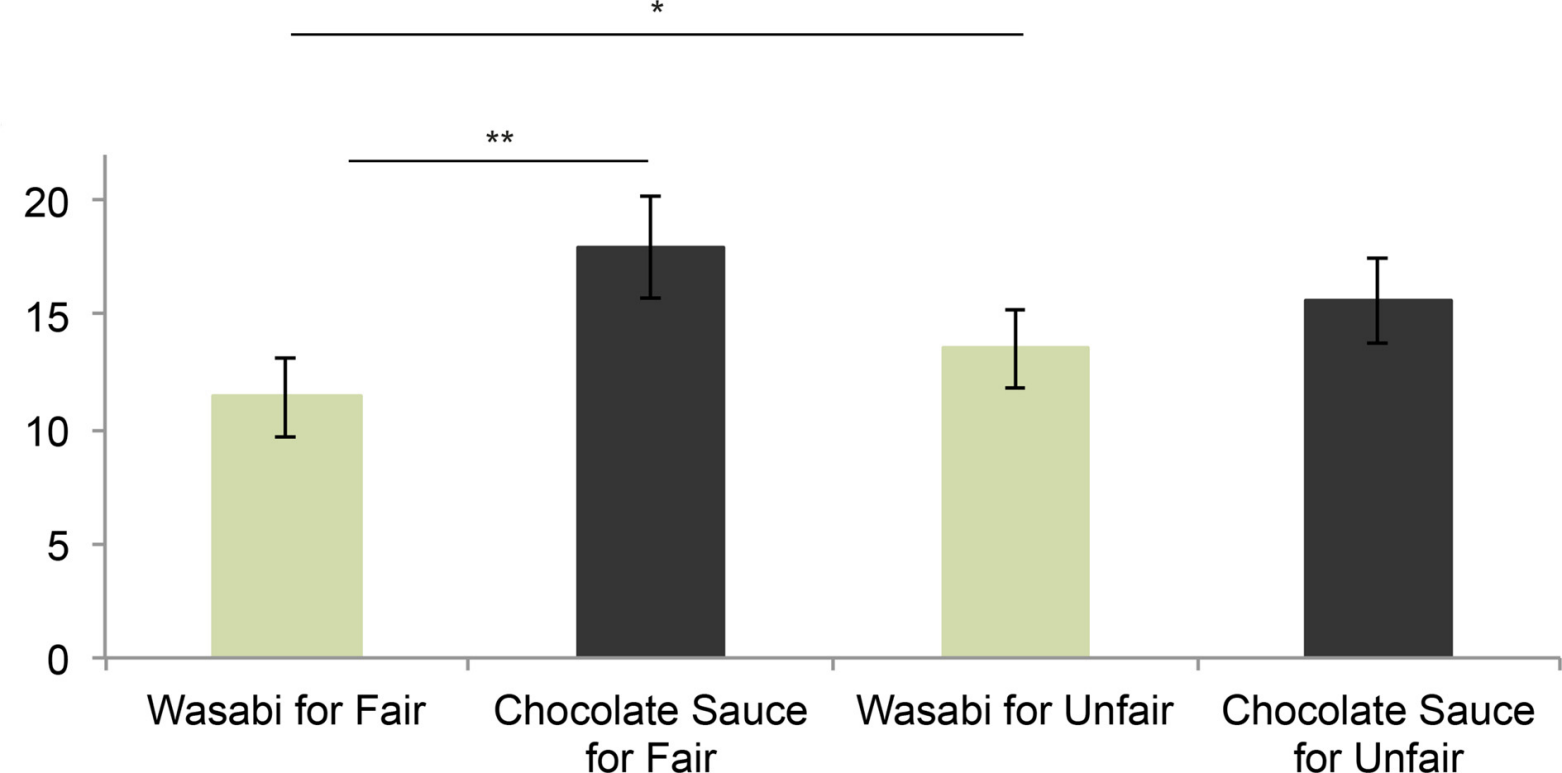
Amount of wasabi and chocolate sauce allocated to the fair and unfair other. Graph depicts means and 2 standard errors of the mean. **p < .01; *p < .05.

A last issue addressed in our paradigm concerns two potential ways of reducing punishment and retaliation behavior after anger elicitation. First, we tested whether the instruction that asked participants to focus on the positive qualities as opposed to the physical appearance of the others prior to the task would influence subsequent punishment behavior in the IG (prediction 4a). Contrary to our expectations, we observed no significant effect (see above). Second, we examined carry-over effects between the IG and behavior in a new context–i.e., the subsequent food allocation task (question 4b). To this end, we conducted two analyses. A repeated measures within-subject MANOVA was performed with the factor other (fair and unfair) and the dependent variable allocated food (chocolate sauce as desirable food and wasabi as undesirable food). This analysis revealed an effect of the factor "other" with a medium effect size (*F(2*,*38)* = 3.33, *p* < .05, *ƞ*^*2*^ = .15; Fig 5). A follow-up univariate ANOVA showed that participants allocated more wasabi to the unfair other than to the fair other (*F*(1,39) = 6.37, *p* < .05). Moreover, paired *t*-tests revealed that participants allocated more chocolate sauce than wasabi to the fair other (*t*(39) = 3.59, *p* < .01). We also tested whether punishment behavior in the food allocation task was correlated with punishment behavior in the high-power phase of the IG. This showed a positive trend for a relation between competitive economic choices and subsequent wasabi allocation to the unfair other (*rs* = .28, *p* = .08), while feedback choices were not related to wasabi allocation (*rs* = -.19, *p* = .25).

## Discussion

Despite the high prevalence of conflict in our society, there has so far been a shortage of tools to empirically study the impact of inter-individual differences on behavioral responses to anger elicitation in controlled laboratory settings. We therefore developed the IG as a paradigm that allows for the assessment of emotional responses to anger elicitation and subsequent other-regarding behavior. First, we validated the IG using a multi-method approach with comprehensive questionnaires, behavioral measures, and physiological measures. In addition, we introduced an extension of a previous measure of aggression–the hot sauce paradigm [43]–that also included a measure of prosocial behavior. Converging evidence from these various measures demonstrates that our paradigm was effective and powerful to modulate emotional and social responses in our participants.

Secondly, we took advantage of this new paradigm to examine several open questions concerning the links between emotions, empathic traits, and social behavior. Our findings reveal that active punishment behavior in the IG was primarily related to the experience of malicious joy, rather than just to negative emotions (such as anger). This extends the notion that malicious joy may be experienced when observing unfair others suffer [16], and that neural activations are increased in reward-related areas during the punishment of unfair others [17]. We also found that empathic traits were related to forgiveness behavior and aggressive behavior in opposing ways. Empathic concern and perspective taking predicted a reduction in punishment behavior (which can be seen as an increase in forgiveness behavior), whereas personal distress predicted aggressive behavior, even towards the fair other. These novel insights may not only help in the understanding of emotional processes underlying punishment behavior, but may also inform interventions that aim at promoting forgiveness behavior.

### Validation of the IG

With regard to the validation of the IG and associated measures (Predictions 1a-1c), we found the following. The efficacy and validity of the IG were confirmed by self-reports of high involvement, while external validity was established by convergent correlations between self-reports of anger and relevant trait questionnaires, such as the STAXI. Among self-reported emotions, the unfair other’s provocation elicited anger, disappointment, and sadness. This result confirms the notion that injustice [55] and insults [56, 57] elicit anger. It also shows that rather than eliciting only anger, competitive economic choices and derogatory feedback messages can induce a wider range of negative emotions, as found in other conditions of injustice [58]. This highlights the importance of studying several emotions in conflict-prone interactions. Consistent with previous findings [59], fair behavior in the IG elicited joy. However, whereas cooperative economic choices were related to joy irrespective of who chose them (the other or the participant), competitive choices elicited a different pattern of emotions, depending on whether they were passively received or actively chosen. Receiving a competitive outcome elicited more anger, more disappointment, and more sadness than making competitive choices for the other. Conversely, actively choosing competitive outcomes for the other was associated with more joy. This finding shows that emotions differ markedly depending on situational variations of power and agency [60] and underlines that a particular kind of joy may contribute to the active punishment of unfair others (for the discussion of malicious joy, see below).

Likewise, measures of arousal revealed that participants reacted to socially relevant situations (cooperative and competitive choices of others) with stronger physiological responses (SCRs) and stronger grip force than they did to control conditions of no social relevance (win and no win conditions). The elevated SCRs for both cooperative and competitive choices of others confirm the sensitivity of arousal to both positive and negative events [30]. In contrast, handgrip force increased when participants received cooperative as compared with competitive distributions. These data are in accord with previous observations that physical effort is sensitive to monetary reward motivation [31, 32], and they extend these findings to the domain of social reward.

On the behavioral level, participants reciprocated the fair and unfair choices of others in the IG, albeit to an attenuated extent compared to the fair and unfair other. Participants could be categorized on the basis of these choices (prediction 1d), in line with the social value orientation framework [61], corresponding to prosocial, sanctioning, or competitive behaviors. Interestingly, participants’ economic and feedback choices in the IG overall were more prosocial than expected by chance distribution. This is in line with previous observations that aggression is not a necessary consequence of provocation, frustration, or anger [15, 62], and raises the question of how inter-individual differences in behavioral responses to anger elicitation can be explained.

### Role of emotions and empathy-related traits

We were able to relate social behavior in the IG with both implicit (prediction 2a) and explicit (question 2b) measures of experienced emotions. By recording spontaneous facial expressions, we found that more frequent smiles during the low power phase of the IG predicted subsequent forgiveness behavior. This result extends the notion that positive emotions foster cooperation while negative emotions increase competition [12, 13] by showing that such effects can be unveiled through spontaneous facial expressions preceding social behavior. On the other hand, although participants reported more anger when receiving unfair outcomes and when actively engaging in punishment behavior, state anger in both cases was not related to participants' propensity for punishment behavior. This result may seem surprising in light of the observed correlation between trait anger and aggression. However, it is in line with previous reports of a weak relationship between self-reported state anger and aggression [63].

Whereas state anger did not correlate with aggression in the current experiment, active punishment behavior in the IG was related to the experience of malicious joy. This finding emphasizes that malicious joy is not only experienced when passively witnessing an unfair other person experiencing pain [16], but that it may also be linked to active punishment behavior. This observation broadens Berkowitz’s hypothesis by underlining that in addition to negative emotions such as anger, reward-related emotions such as malicious joy can play a role in active punishment. In contrast, we observed that the negative emotion of regret was related to a reduction in both prosocial and punishment behavior, further arguing against the simple notion that unpleasant emotions are linked to aggression [2]. Instead, it seems that the regret could rather lead to a disengagement from the associated behavior.

With regard to the role of empathy-related traits (prediction 3a), we found that forgiveness behavior was predicted by empathic concern and perspective taking. In contrast, individual proneness to empathic distress was related to increased aggressive behavior, even towards the fair and innocent other. This result limits and extends several previous findings. First, our data underline that empathic traits are not necessarily linked to reduced aggressive behavior [25], but that different empathic traits can be related to aggressive behavior in opposing ways. Previous studies on prosocial behavior reported that empathic distress was associated with reduced helping [3], while empathic concern increased helping [3, 4]. We show that a similar distinction applies to antisocial behavior following anger induction.

Furthermore, the observed relation between perspective taking and conciliatory behavior extends previous findings on the beneficial impact of perspective taking skills onto negotiation outcomes [64]. In addition to the beneficial effects of perspective taking on altruistic behavior and helping behavior [24], perspective taking in our experiment was positively related to the engagement in forgiveness behavior. Overall, these results underline the importance of considering empathy as a multidimensional construct, especially when studying social interactions.

Interestingly, derogatory feedback messages to the fair and unfair other were not only predicted by personal distress, but also by the tendency for behavioral inhibition, a construct related to anxiety and submissiveness [51]. Although this is in contrast to the previous observation that behavioral inhibition is negatively related to aggression [50], the current data suggest that the proneness to experience aversive social feelings is related to augmented antisocial behavior.

Given observations that positive affect is associated with more cooperation [1,12,13], we hypothesized that focusing on positive qualities of the others might reduce subsequent punishment behavior (prediction 4a). This hypothesis was not confirmed. This might be due to the temporal delay between this description exercise (prior to the IG) and the opportunity for punishment behavior (in the second part of the IG). Future studies should test whether focusing on the others' positive qualities at a different time or using a different exercise can affect social behavior.

Finally, to test the hypothesis that “settling the score” may decrease subsequent aggression [27] (question 4b), we also examined whether punishment behavior in the Inequality Task predicted the allocation of undesirable spicy food (wasabi) to the unfair other in a subsequent task. Our data revealed a trend for economic punishment to predict the amount of wasabi allocated to the unfair other. This finding indicates that an opportunity for "settling the score" in the same situation did not diminish subsequent punishment. Rather, punishment behavior in the first situation (IG) was related to augmented antisocial behavior in the subsequent food allocation situation. Taken together, in the present experiment, neither focusing on other´s positive qualities nor “settling the score” reduced subsequent punishment.

### Limitations and future directions

This paper introduces the IG as a novel tool to study behavioral reactions linked to anger induction in controlled laboratory settings. We validate this task in a multi-method study and reported predictors of social behavior at the level of emotions and empathy-related personality traits. Despite these assets, there are several limitations to the results, which might be addressed in future studies. A central limitation is that–apart from the manipulation of the others’ fairness in the IG–most reported findings are correlational. Future studies are needed to test for causal relationships and between personality traits, emotions, and social behavior. Moreover, because of previously reported sex differences in aggression [35, 36], as well as emotional responses to unfairness [16], the present study recruited male participants only. Future studies are needed to test whether similar relations hold true in females.

Another limitation is linked to the recording of subjective feelings after the IG, which was done to avoid interference of self-reports on behavior [54]. The temporal delay between provocation and self-reports may have distorted participants’ recollection of their feelings during the IG. In addition, self-reports of participants’ feelings were collected only in response to reciprocal situations (i.e., cooperative economic choices and nice feedback for the fair other and competitive economic choices and derogatory feedback for the unfair other). To obtain finer-grained insights, future studies should assess a broader range of situations. Although the present study took into account physiological markers related to arousal, it would be interesting to elucidate the neural substrates of anger and to dissociate them from aggressive behavior. In this context, it will also be important to test which neural functions control social behavior and foster forgiveness in response to provocations.

## Supporting Information

S1 File Text A (including Supporting Material and Methods, Supporting Results, Supporting Discussion), Supporting Figure and Tables. (PDF)
