# Supplementary material for: The Impact of Emotions and Empathy-Related Traits on Punishment Behavior: Introduction and Validation of the Inequality Game
Source: PLoS One. 2016 Mar 15;11(3):e0151028. doi: 10.1371/journal.pone.0151028 (PMC4792486; doi:10.1371/journal.pone.0151028)
Supplement: S1 File — (PDF) [file pone.0151028.s001.pdf]

S1 FILE

The Impact of Emotions and Empathy-Related Traits on Punishment Behavior: Introduction and Validation of the Inequality Game

Olga M. Klimecki[1,2], Patrik Vuilleumier[1,3], David Sander[1,2]

[1] Swiss Center for Affective Sciences, University of Geneva, Geneva, Switzerland

[2] Laboratory for the Study of Emotion Elicitation and Expression, Department of Psychology, University of Geneva, Geneva, Switzerland

[3] Laboratory for Behavioral Neurology and Imaging of Cognition, Department of Neuroscience, Medical School, University of Geneva, Geneva, Switzerland

Text A

Supporting Material and Methods

Procedure

In each experimental session, the participant and the two confederates were met by the experimenter at the entrance door of the laboratory. They were then led to a common room with another alleged experimenter. Participants were informed that the experiment consisted of two stages: an economic interaction game and a food evaluation task. They then read and signed the consent form. Photographs of all alleged participants were taken to be used in the economic interaction game. Subsequently, a questionnaire about taste preferences was filled in. At this point, both confederates stated that they strongly dislike spicy food and feared eating it in the food evaluation part. Finally, all three alleged participants wrote a brief description of the other two participants. Half of the participants were asked to describe the positive personal qualities they had noticed in the others and the other half to describe the physical appearance of the others.

Next, the participant and the two confederates were led into different rooms of the laboratory. The participant was guided into the psychophysiology room, while one confederate was allegedly tested in the eye-tracker room and the other confederate in the behavioral testing room. In the psychophysiology room, participants read written instructions about the economic interaction game. Their comprehension of the task was probed and questions regarding the task were answered by the experimenter. Participants were first prepared for psychophysiological recordings and then calibrated a handgrip device that served for response selection. To familiarize participants with the economic interaction game, we did a test phase in which the participant experienced both the low and high power conditions. This test phase was always allegedly played with the fair other. Both confederates were counterbalanced to the fair and unfair condition and were not aware of their role (fair or unfair) in the subsequent IG (to avoid any bias due to an uncontrolled factor

from first impressions). Before playing two phases of the economic interaction game, participants were reminded that one trial from each phase would be randomly selected and used as a real payoff at the end of the experiment, resulting in a potential additional gain from 0 CHF to 20 CHF. Each phase of the IG lasted about 12 minutes and was accompanied by concurrent recordings of facial expressions by a camera mounted on the computer screen. Upon completion of the IG, participants filled in a questionnaire about their emotional reactions to the different situations encountered during the game (economic and feedback exchanges) and then completed the food allocation task. At the end of the experiment, participants filled in the debriefing questionnaire, were thanked, probed for suspicion, fully debriefed, and received 25 CHF for their participation.

Measures

Questionnaires

The STAXI allowed us to assess whether state anger elicited by the Inequality Game (IG) was related to trait anger and anger expression. In the STAXI, trait anger consists of the subscale angry temperament (T, experience of anger without specific provocation) and the subscale angry reaction (R, angry reactions to frustration). In the STAXI, anger expression (AX) consists of four subscales: anger-in (AX/In), anger-out (AX/Out), and two anger control subscales (AX/Con-In and AX/Con-Out). “In” concerns anger experiences that are not displayed, “Out” relates to outward expressions of anger experiences, and “Con” relates to the energy used to control and reduce the respective anger experiences. The AQ, which served to validate the IG as a means of inducing aggressive behavior, contains four subscales: anger, physical aggression, verbal aggression, and hostility. The Psychopathy Scale measures primary psychopathy (i.e., emotional-interpersonal tendencies related to narcissism and social dominance) and secondary psychopathy (defined as social deviance, such as aggressiveness and impulsiveness). The BIS/BAS consists of four subscales: BIS

(reactions to anticipated punishment), BAS reward responsiveness (positive responses to occurrence or anticipation of rewards), BAS fun seeking (desire for new rewards), and BAS drive (persistent pursuit of desired goals). The subscales of the Interpersonal Reactivity Index (IRI) are perspective taking, empathic concern, personal distress, and fantasy.

Food Allocation Task

For this task, participants were seated at a table in their experimental room and were shown a food evaluation questionnaire, four plastic spoons (to taste four different food items), a glass of water (to rinse the mouth between the different food items), a nontransparent box (containing jars filled with the food items “C” and “D” for the others), four transparent sealable plastic containers (in which to place the food for the other participants), two metal spoons (to handle the food items for the others), and a pen to label the plastic containers (with “C” and “D”). Participants were told that upon receipt of the four food items, each participant had to eat the entire quantity of food in each container before evaluating each sample's taste. Participants were told that in order to blind the experimenters to the conditions, each participant was to allocate two food items (allegedly denoted by letters ranging from A to F) from a nontransparent box into sealable plastic containers and nontransparent boxes for the other two participants. Finally, the formerly completed food preference questionnaires of the fair and unfair other were shown to participants. These questionnaires (ranging from 0, do not like at all, to 10, like extremely) clearly indicated a dislike of spicy food (rating of 1) and a preference for sweet food (rating of 9) for both others. Participants were then left alone to assign the two food items (spicy wasabi sauce and sweet chocolate sauce) to the others. The chocolate sauce was slightly diluted with water to obtain the same specific weight as the wasabi sauce. This ensured a corresponding volume-weight ratio for both substances.

Supporting Results

Validation of the IG

To determine whether the other's fairness during the IG had an impact on subsequent person evaluation, we computed a repeated measures multivariate analysis of variance (MANOVA) with the within-subject factor other (two levels: fair and unfair) and the evaluations of the other as fair, reliable, agreeable, and good looking as dependent variables. As expected, the others' behavior during the IG had a large effect on these ratings ($F(4,36) = 15.64$, $p < .001$, $\eta^2 = .64$). Univariate tests confirmed the effect of the fairness manipulation on person evaluation. Although confederates were counterbalanced to the fair and unfair condition, the fair other was consistently evaluated as more fair, reliable, and agreeable than the unfair other (all $F(1,39) \geq 45.07$, all $p < .001$; Table S6). In addition, there was a trend for the fair other to be evaluated as more good looking than the unfair other ($F(1,39) = 3.25$, $p = .08$).

Fair and unfair behavior elicit different patterns of emotions

To test whether the fair and unfair others' economic choices and feedback behavior in the low power phase of the IG elicited different feelings, we carried out a 2 x 2 repeated measure MANOVA with the within-subject factor event type (economic choice and feedback choice) and other (fair and unfair). The dependent variables were self-reports of joy, anger, disappointment, and sadness. We found a significant main effect of other ($F(4,36) = 49.72$, $p < .001$, $\eta^2 = .85$) and event type ($F(4,36) = 7.82$, $p < .001$, $\eta^2 = .47$), as well as an interaction between event type and other ($F(4,36) = 8.39$, $p < .001$, $\eta^2 = .48$). All effect sizes were large, suggesting that the manipulation had strong effects on participants' feelings. Univariate tests revealed that the effect of other was significant for all dependent variables (all $F(1,39) \geq 24.66$, all $p < .001$). As depicted in Fig 2, economic and feedback choices of the fair other

elicited higher self-reports of joy, whereas the behavior of the unfair other induced stronger feelings of disappointment, anger, and sadness. In terms of event type, economic choices elicited more anger ($F(1,39) = 4.79$, $p < .05$) and disappointment ($F(1,39) = 28.17$, $p < .001$) than did feedback choices. The interaction between event type and other was significant for disappointment ($F(1,39) = 16.93$, $p < .001$) and marginally significant for anger ($F(1,39) = 3.9$, $p = .06$). Follow-up *t*-tests revealed that there was no effect of event type on self-reported feelings of disappointment or anger in response to fair behavior ($t(39) = .27$, $p = .79$ and $t(39) = .26$, $p = .8$, respectively), whereas more disappointment ($t(39) = 5.05$, $p < .001$) and anger ($t(39) = 2.24$, $p < .05$) were reported in reaction to the unfair other's economic as compared with feedback choices.

State anger is predicted by trait anger, aggression, and psychopathy

To assess the external validity of anger induction through the IG, we computed correlations between trait questionnaires and self-reports of anger in response to the others' economic and feedback choices (low power phase). There was a trend for a correlation between total trait anger (STAXI) and state anger in response to competitive economic choices ($rs = .3$, $p = .06$). Further analyses revealed that the subscale angry temperament predicted anger responses in relation to unfair economic choices ($rs = .33$, $p < .05$). Anger at competitive economic splits was also predicted by secondary psychopathy ($rs = .4$, $p < .05$) and total trait aggression (AQ; $rs = .41$, $p < .01$). The latter relation was significant for the AQ subscales physical aggression, anger, and hostility (all $rs \geq .32$, all $p < .05$). These correlations of medium effect size confirm the validity of the IG as a tool for anger induction. In fact, trait anger (STAXI) was correlated with anger feelings only in response to competitive economic choices and with no other feelings in response to economic choices (all other $rs \leq .23$, $p \geq .15$). However, aggression (total AQ) also predicted disappointment in response to competitive economic choices ($rs = .42$, $p < .01$). This relation was significant for the

subscales anger and hostility (both *rs* ≥ .37, both $p < .05$). Finally, secondary psychopathy also predicted disappointment and sadness in response to other's competitive choices (both *rs* ≥ .36, both $p < .05$). None of the expected measures (STAXI trait, AQ total, primary and secondary psychopathy) predicted anger in response to other's derogatory feedback (all *rs* ≤ .25, all $p \geq .13$). This result indicates that although self-reported feelings were similar for economic choices and feedback behavior of the other, the relationship with trait questionnaires was more pronounced for unfair economic choices. This might be due to higher self-reports of anger and disappointment in responses to unfair economic choices as compared with derogatory feedback messages.

Participants' preferences for prosocial, sanctioning, or competitive behavior are reflected in other's economic outcomes

In our sample, 21 of 40 participants were identified as prosocial, 11 as sanctioning, and 7 as competitive. There was one participant who did not clearly fall into any of these three categories, as he chose as many cooperative as competitive outcomes for the fair and unfair other. To examine how participants' behavior affected the others' economic outcomes, we conducted a MANOVA with the between-subject factor economic preference (three levels: prosocial, sanctioning, and competitive). The dependent variables were the hypothetical overall gains of the participant, the fair other, and the unfair other (all in the high power phase). Results revealed a large effect of economic preference ($F(6,70) = 87.28$, $p < .001$, $\eta^2 = .88$). As expected, univariate ANOVAs showed that behavioral preference had no impact on participants' own gain ($F(2,36) = 1.31$, $p = .28$), whereas the effects were significant for the fair and unfair others' gains (both $F(2,36) \geq 105.07$, both $p < .001$; Fig 3D). Follow-up independent *t*-tests revealed that prosocial and sanctioning participants allocated high monetary outcomes to the fair other ($t(30) = .45$, $p = .66$), whereas the unfair other received less in interactions with sanctioning as compared with prosocial participants ($t(30)$ =

15.69, $p < .001$). The comparison between prosocial and competitive participants revealed that both the fair and unfair other had lower economic gains in interactions with competitive as compared with prosocial participants (both $t(26) \geq 12.91$, both $p < .001$). Finally, fair others received higher economic gains when faced with sanctioning as compared with competitive participants ($t(26) = 14.74$, $p < .001$), whereas the preferences for sanctioning or competitive behavior made no difference to the unfair other's economic gain ($t(16) = .98$, $p = .34$).

Personality trait differences for distinct classifications

As a next step, we tested whether participants' classifications were reflected in personality traits as measured by questionnaires. ANOVAs with the between-subject factor economic preference (three levels: prosocial, sanctioning, and competitive) revealed significant differences of medium effect size for total trait anger (STAXI; $F(2,36) = 3.91$, $p < .05$, $\eta^2 = .18$) and for angry reactions to provocation (STAXI-R; $F(2,36) = 6.73$, $p < .01$, $\eta^2 = .27$), as well as a trend for an effect of perspective taking (IRI; $F(2,36) = 2.46$, $p = .1$, $\eta^2 = .12$). Prosocial participants were lower in total trait anger than were competitive participants ($p < .05$) and there was a similar tendency in the comparison of prosocial participants with sanctioning participants ($p = .08$). Similarly, anger reactions to provocation (STAXI-R) were lowest in prosocial participants as compared with sanctioning ($p < .05$) and competitive participants ($p < .01$). Conversely, there was a trend for the prosocial participants to score higher on perspective taking than there was for the sanctioning ($p = .11$) or competitive participants ($p = .07$).

Classification of Feedback Choices

With regard to feedback choices, 34 of 40 participants gave mainly nice feedback to both the fair and unfair other, whereas 4 participants sent mainly nice feedback to the fair other and derogatory feedback to the unfair other. Only one participant gave mainly

derogatory feedback to both the fair and unfair other. Finally, one participant sent predominantly nice feedback to the unfair other and derogatory feedback to the fair other.

Arousal and effort are sensitive to social situations

Arousal

In order to assess participants' sympathetic arousal responses to the fair and unfair other's economic and feedback choices in the IG, we conducted a 2 x 3 repeated measures ANOVA with the within-subject factors other (fair and unfair other) and three economic outcome conditions: i) choice of other, ii) win control, and iii) no win control (Fig A). The dependent variable was the skin conductance response (SCR) to the display of the other's economic choice on the computer screen. The main effect of outcome condition was significant with a medium effect size ($F(2,68) = 7.22$, $p < .01$, $\eta^2 = .18$). Follow-up tests revealed that deliberate choices of the other ($M = .15$, $SD = .14$) elicited a higher SCR than did win ($M = .07$, $SD = .11$, $p < .05$) or no win ($M = .05$, $SD = .09$, $p < .01$) control conditions. Neither the main effect of other nor the interaction between other and outcome condition was significant ($F(1,34) = 0.07$, $p = .8$ and $F(2,68) = 0.22$, $p = .81$, respectively). To test for differences in SCR with respect to nice and derogatory feedback, we computed a paired *t*-test. In parallel with the results for economic choices, there was no significant difference in SCR in response to nice feedback ($M = 0.08$, $SD = 0.14$) and derogatory feedback ($M = 0.13$, $SD = 0.21$; $t(34) = 1.63$, $p = .11$). In summary, SCRs in our experiment were not sensitive to reward, either in terms of monetary outcomes or in terms of feedback messages. However, participants showed increased arousal responses to socially relevant situations; this result emphasizes the responsiveness of the physiological system to relevant events involving deliberate social decisions.

Effort

In order to implicitly assess physical effort, we measured the handgrip force that participants used when making their choices during the IG. We conducted, in parallel with the SCR analysis, a 2 x 3 repeated measures ANOVA with the within-subject factors other (fair and unfair other) and three economic outcome conditions: i) choice of other, ii) win control, and iii) no win control. The dependent variable was the handgrip force participants used to choose the payoff column. Although the effect of other was not significant ($F(1,39) = 0.74$, $p = .4$), we found a substantial main effect of outcome condition ($F(2,78) = 20.95$, $p < .001$, $\eta^2 = .35$) and an interaction of medium effect size between other and outcome condition ($F(2,78) = 28.51$, $p < .05$, $\eta^2 = .09$). As depicted in Table B, participants used a stronger handgrip force when reacting to the other's deliberate choice as compared with the win and the no win control condition (both $p < .001$). The overall difference between the win and no win control condition was not significant ($p = .19$). In terms of the interaction, follow-up paired *t*-tests revealed that participants reacted to fair deliberate choices ($M = 27.41$, $SD = 19.77$) with stronger handgrip force than they did to unfair deliberate choices ($M = 26.33$, $SD = 18.59$; $t(39) = 2.74$, $p < .01$). However, the fairness of the other had no effect on handgrip force in the win or the no win control conditions ($t(39) \leq 1.39$, $p \geq .17$).

Taken together, higher arousal and stronger physical effort in response to socially relevant situations indicate that socially relevant situations lead to the mobilization of resources, be it on the physiological level or on the level of muscular activity. In addition, participants used more effort in response to fair as compared with unfair economic distributions, corroborating previous results on the sensitivity of physical effort to reward motivation [1, 2].

Aggression is Predicted by Trait Anger and Psychopathy

In terms of personality traits, anger (STAXI total; e.g., “I have a fiery temper”) predicted the degree of competitive economic choices for the unfair other (*rs* = .33, *p* < .05). In particular, the subscale angry reactions to provocation (STAXI-R; e.g., “It makes me furious when I am criticized in front of others”) predicted competitive economic choices towards the fair and unfair other (both *rs* ≥ .33, both *p* < .05) as well as derogatory feedback towards the fair and unfair other (both *r* ≥ .36, both *p* < .05). Conversely, control of outward anger expression (STAXI AX/Con-Out) predicted cooperative economic choices (*rs* = .37, *p* < .05) and nice feedback (*r* = .43, *p* < .01) for the unfair other. Primary psychopathy, which is a measure of social dominance (e.g., “Looking out for myself is my top priority”), predicted competitive economic choices for the unfair other (*rs* = .41, *p* < .01), whereas secondary psychopathy, which is a measure of antisocial behavior (e.g., “When I get frustrated, I often “let off steam” by blowing my top”), predicted derogatory feedback towards the unfair other (*r* = .4, *p* < .05). To our surprise, neither the total aggression questionnaire (AQ) nor its subscales predicted behavior in economic and feedback choices and in food allocations, both towards the fair and unfair other (all *rs* ≤ .25, all *p* ≥ .13). This might be explained by the items of the AQ pertaining to more severe cases of aggression (e.g. “Given enough provocation, I may hit another person”) than the behavioral reactions implemented in the IG. In terms of correlations with food allocation, there was a trend for anger (STAXI total) to be related to wasabi allocations towards the fair other (*rs* = .29, *p* = .07) and a trend for outward anger expression (STAXI AX/Out) to be positively related to wasabi allocation for the unfair other (*rs* = .28, *p* = .08).

Opposing Patterns of Emotions for the Reciprocation of Fair and Unfair Behavior

We tested whether participants experienced different emotions when choosing cooperative economic splits and nice feedback for the fair other as opposed to competitive economic splits and derogatory feedback for the unfair other. For economic choices, this was done by means of a repeated measures MANOVA with the within-subject factor economic situation (two levels: cooperative economic choice for the fair other or competitive economic choice for the unfair other) and the dependent variables anger, joy, disappointment, regret, culpability, malicious joy, generosity, and sadness in that situation. We observed a substantial effect of fairness ($F(8,17) = 11.68$, $p < .001$, $\eta^2 = .85$). As depicted in Table C the choice of competitive economic splits for the unfair other as compared with cooperative economic choices for the fair other was associated with more anger, regret, culpability, and malicious joy. Conversely, choosing a cooperative economic split for the fair other was associated with stronger feelings of generosity. To examine emotional experiences during feedback choices, we performed a repeated measures MANOVA with the within-subject factor feedback situation (nice feedback for the fair other and derogatory feedback for the unfair other) and the dependent variables anger, joy, disappointment, regret, culpability, malicious joy, generosity, and sadness. There was a large overall effect of feedback situation ($F(8,21) = 16.65$, $p < .001$, $\eta^2 = .86$). Univariate ANOVAs confirmed the effect for all dependent variables (Table D). Giving derogatory feedback to the unfair other was thus associated with stronger anger, disappointment, regret, culpability, malicious joy, and sadness, whereas giving nice feedback to the fair other was related to stronger feelings of joy and generosity.

The Effect of Power on Self-Reported Feelings

To test for different feelings in response to the others' as opposed to participants' own behavior, we performed repeated measure MANOVAs with the within-subject factor power (two levels: low and high power phase) and the dependent variables joy, anger,

disappointment, and sadness. In order to conserve sample sizes (participants varied in their behavioral choices – not all participants engaged in all kinds of behaviors), this was done separately for ratings related to cooperative economic choices (n = 35), competitive economic choices (n = 30), nice feedback (n = 40), and derogatory feedback (n = 29). Although there was no effect of power on ratings related to cooperative economic behavior ($F(4,31) = 0.8$, $p = .54$), power had a substantial impact on ratings related to competitive economic behavior ($F(4,26) = 15.81$, $p < .001$; $\eta^2 = .71$). Univariate tests confirmed that this effect was highly significant for all dependent variables (all $F(1,29) \geq 17.66$, all $p < .001$). As depicted in Table E, stronger feelings of joy were reported in relation to own unfair competitive behavior, whereas higher feelings of anger, disappointment, and sadness were experienced in response to another's competitive economic choices. Performing equivalent analyses for feedback choices revealed that there was no effect of power on nice ($F(4,36) = 1.15$, $p = .35$) or derogatory feedback ($F(4,25) = 2.07$, $p = .12$). These data indicate that cooperative monetary choices, whether passively received or actively chosen, elicited similar emotional reactions (high joy as well as low anger, disappointment, and sadness). Conversely, emotional experiences related to competitive economic choices differed markedly: passively receiving a competitive monetary distribution elicited more anger, disappointment, and sadness, whereas stronger joy was experienced when actively punishing the unfair other.

Relation between Emotions, Empathic Traits, Person Evaluation, and Social Behavior

We next examined how emotional expressions during the IG were related to the evaluation of the others. Smiling during the low power phase positively predicted the subsequent evaluation of the unfair and the fair other as good looking (both $rs = .37$, *both* $ps < .05$) and the evaluation of the unfair other as agreeable ($r = .38$, $p < .05$). Conversely,

frowning during the low power phase negatively predicted the rating of the unfair other as agreeable (*rs* = -.35, $p < .05$) and more marginally as good looking (*rs* = -.31, $p = .06$). All other correlations were not significant (all other *rs* ≤ .23, all other $p \geq .16$).

The average intensity of smiling behavior was also correlated positively with perspective taking (IRI PT; $r = .37$, $p < .05$) and negatively with anger expression (STAXI-AX; $r = -.33$, $p < .05$), while there was a trend for a positive relation with anger control (STAXI AX/Con-In; $r = .32$, $p = .06$). Conversely, frowning during the low power phase was negatively predicted by perspective taking as measured with the IRI (*rs* = -.38, $p < .05$), indicating that people with a higher capacity for perspective taking frown less frequently. No other relations with trait variables were found (all other $r \leq .25$, all other $p \geq .13$).

Next, we examined whether empathic dispositions predicted emotional reactions to others' unfair behavior in the low power phase ($n = 40$) and emotions associated with own unfair behavior in the high power phase ($n = 30$). We found that the disposition for perspective taking was negatively associated with disappointment when the participants were faced with unfair economic choices (*rs* = -.32, $p < .05$), with a similar trend for anger (*rs* = -.3, $p = .06$). Moreover, perspective taking was positively related to joy when the participants were faced with unfair choices (*rs* = .35, $p < .05$). During the high power phase, empathic concern was positively related to self-reports of anger and sadness during own unfair choices (both *rs* = .4, both $p < .05$).

Finally, we tested how empathy-related traits predicted person evaluation. Overall empathy (IRI total) positively correlated with the evaluation of the unfair other as fair (*rs* = .37, $p < .05$). It was the subscale perspective taking that positively predicted ratings of the unfair other as fair (*rs* = .32, $p < .05$) and as agreeable (*rs* = .49, $p < .01$). Person evaluation also correlated with behavior during the IG. Participants who perceived the unfair other as more fair or agreeable gave nicer feedback to the unfair other (both $r \geq .38$, both $p < .05$). Similarly, participants who perceived the fair other as more fair, agreeable, and reliable chose nicer

feedback and more cooperative economic outcomes for the fair other (all *rs* ≥ .35, all $p \leq .05$). All other correlations between person evaluation and behavior in the high power phase of the IG or the food allocation task were not significant.

Supporting Discussion

The Link between Emotions, Empathic Personality Traits, and Social Behavior

With regard to emotions, positive affect expressed in smiles predicted a more favorable impression of the fair and unfair other, whereas frowning predicted more unfavorable evaluations of the unfair other. With regard to empathy-related traits, perspective taking was associated not only with increased forgiveness behavior, but also with self-reports of more positive emotions (joy) and less experiences of negative emotions (disappointment and anger) when participants were faced with provocations. Similarly, empathic concern tended to predict more positive emotions (joy) during derogatory feedback. Although such an increase in positive emotions in response to provocation may be puzzling at first sight, it ties in with previous observations that compassion (which is conceptually similar to empathic concern) is related to positive emotions in general [3]. In fact, the current data extend this notion in two ways: first, by showing that the link between empathic concern and positive emotions also applies to situations involving provocation and second, by observing a similar relation for perspective taking.

Interestingly, a different pattern of emotions was associated with the participant's own competitive choices. Here, empathic concern was correlated with

stronger anger and sadness in relation to active economic punishment behavior, whereas perspective taking showed a tendency to be negatively related to malicious joy. In addition, perspective taking predicted more favorable evaluations of the unfair other and more positive facial expressions – two constructs that were in turn related to a reduction in punishment behavior in the IG. In other words, our study shows that perspective taking skills are related to a wide range of social processes, including facial expressions, emotional experiences, person perception, and social behavior.

Figures and Tables

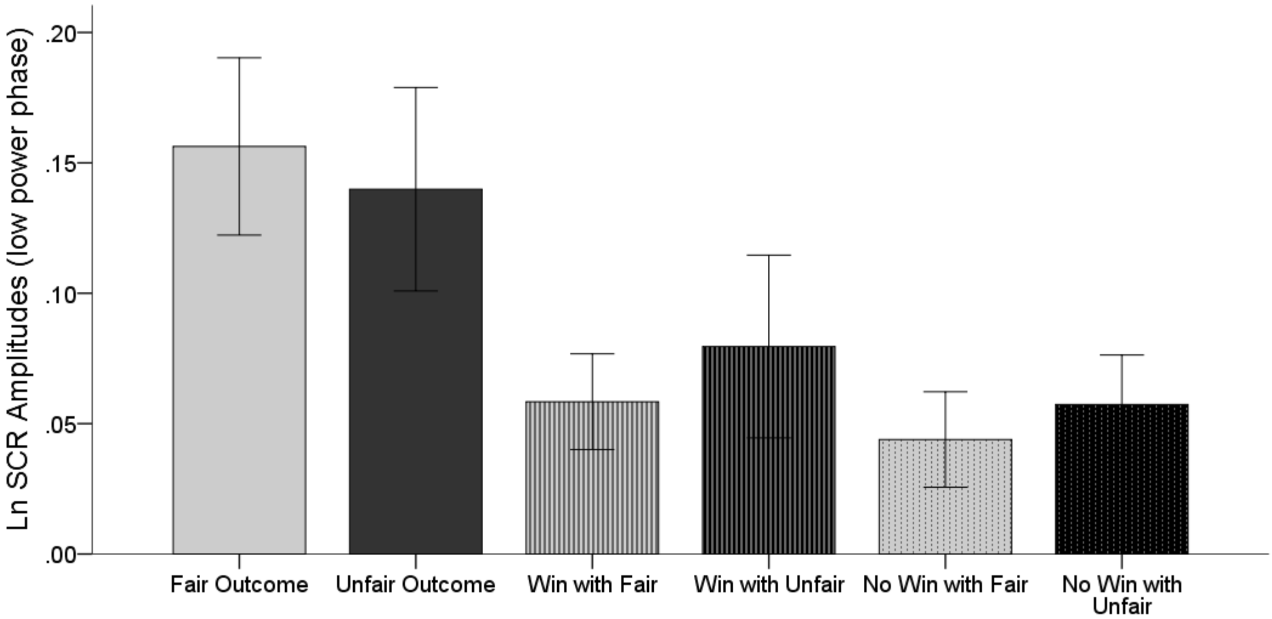

Fig A. Log-transformed skin conductance response (SCR) amplitudes for different outcome frames in the low power phase of the Inequality Game. Charts depict means and 2 standard errors of the mean. Analyses and results are described in the text.

Table A. Summary of psychometric data.

	M	*SD*
Depression (BDI)	5.90	4.89
Alexithymia (TAS)	51.43	5.36
Trait Anger (STAXI)	20.80	4.33
Angry Temperament	6.98	2.11
Angry Reaction	10.18	2.41
Anger Expression/Control (STAXI)	37.98	11.22
External Anger Expression	16.70	3.89
Internal Anger Expression	18.45	3.73
External Anger Control	23.58	4.78
Internal Anger Control	21.60	4.93
Aggression (AQ)	63	14.7
Physical Aggression	17.63	5.46
Verbal Aggression	14.68	3.85
Anger	15.00	4.64
Hostility	15.70	5.13
BAS Drive	12.45	1.95
BAS Fun Seeking	11.80	1.84
BAS Reward Responsiveness	18.03	1.72
BIS	18.48	3.84
Primary Psychopathy	32.73	7.01
Secondary Psychopathy	19.43	4.19
Empathy (IRI)	65.85	12.16
Perspective Taking	17.43	4.60
Fantasy	17.63	5.76
Empathic Concern	18.95	5.34
Personal Distress	11.85	4.06

Note. AQ, Aggression Questionnaire; BAS, Behavioral Activation System; BDI, Beck's Depression Inventory; BIS, Behavioral Inhibition System; IRI, Interpersonal Reactivity Index; STAXI, State-Trait Anger Expression Inventory; TAS, Toronto Alexithymia Scale.

Table B. Handgrip force (% of maximum) in response to other's choice in the low power phase.

Outcome Condition	*M*	*SD*
**Other's Choice**	26.87	19.15
**Win**	24.07	16.88
**No Win**	23.62	16.73

Table C. Self-reported feelings related to the choice of cooperative economic splits for the fair other as opposed to the choice of competitive economic splits for the unfair other during the high power phase.

Self-reported Feeling	Cooperative Choice *M (SD)*	Competitive Choice *M (SD)*	*p*
**Anger**	0.32 (1.41)	2.04 (2.87)	<.05
**Joy**	5.76 (2.82)	5.56 (3.03)	= .8
**Disappointment**	0.32 (1.22)	1.04 (2.13)	= .14
**Regret**	0.6 (1.87)	2.36 (3.17)	<.05
**Culpability**	0.12 (0.44)	2.0 (2.68)	<.01
**Malicious Joy**	0.16 (0.63)	3.44 (3.57)	<.001
**Generosity**	6.48 (2.58)	0.4 (1.61)	<.001
**Sadness**	0.32 (1.07)	0.68 (1.73)	= .4

Note. n = 25.

Table D. Self-reported feelings related to the choice of nice feedback for the fair other as opposed to derogatory feedback for the unfair other during the high power phase.

Self-reported Feeling	Nice Feedback Choice: *M (SD)*	Derogatory Feedback Choice: *M (SD)*	*p*
**Anger**	0.35 (0.19)	4.69 (3.04)	<.001
**Joy**	7.21 (2.58)	1.14 (2.25)	<.001
**Disappointment**	0.17 (0.6)	4.35 (3.5)	<.001
**Regret**	0.31 (0.81)	1.62 (2.54)	<.05
**Culpability**	0.17 (0.38)	1 (1.51)	<.01
**Malicious Joy**	0.17 (0.54)	1.72 (2.98)	<.01
**Generosity**	6 (3.33)	0.21 (0.56)	<.001
**Sadness**	0.1 (0.31)	2.04 (2.87)	<.01

Note. n = 29.

Table E. Self-reported feelings in relation to other's as opposed to own competitive economic choices.

Feeling	Agent	*M*	*SD*
**Joy**	Other	1.13	2.36
	Self	5.9	2.96
**Anger**	Other	4.73	3.45
	Self	1.8	2.71
**Disappointment**	Other	6.13	3.38
	Self	0.87	1.98
**Sadness**	Other	3.07	3.1
	Self	0.57	1.59

Note. n = 30.

Table F. Evaluation of the fair and unfair other.

Evaluation	Other	*M*	*SD*
**Fair**	Fair	7.48	1.9
	Unfair	3.65	2.65
**Agreeable**	Fair	7.35	2.06
	Unfair	3.85	2.71
**Good looking**	Fair	5.03	2.89
	Unfair	4.38	2.56
**Reliable**	Fair	7.1	2.34
	Unfair	3.25	2.41

Supplementary References

1. Pessiglione M, Schmidt, L, Draganski, B, Kalisch, R, Lau H, Dolan, RJ, et al. How the brain translates money into force: A neuroimaging study of subliminal motivation. Science. 2007; **316**(5826): 904-906.
2. Talmi D, Seymour, B, Dayan, P, Dolan RJ. Human pavlovian-instrumental transfer. J Neurosci. 2008;**28**(2): 360-368.
3. Klimecki OM. The plasticity of social emotions. Soc Neurosci. 2015. **10** (5): 466-473.
